# Coronavirus Disease 2019 and Human Reproduction: A Changing Perspective

**DOI:** 10.6061/clinics/2021/e3032

**Published:** 2021-08-27

**Authors:** Luciana C. Delamuta, Pedro A.A. Monteleone, Edson S. Ferreira-Filho, Vanessa Heinrich-Oliveira, José Maria Soares-Júnior, Edmund C. Baracat, Gustavo A.R. Maciel

**Affiliations:** Disciplina de Ginecologia, Departamento de Obstetricia e Ginecologia, Hospital das Clinicas HCFMUSP, Faculdade de Medicina, Universidade de Sao Paulo, Sao Paulo, SP, BR.

**Keywords:** COVID-19, SARS-CoV-2, Infertility, Reproduction, Pregnancy

## Abstract

Since the outbreak of severe acute respiratory coronavirus 2 (SARS-CoV-2), the coronavirus disease 2019 has had a wide range of effects on human health. This paper summarizes the data related to the effects of the SARS-CoV-2 infection on human reproduction.

Both the male and female reproductive tract express high levels of receptors and proteins needed for viral cell entry. There is presently no evidence that gametes are affected by the infection. Male fertility may be temporarily reduced due to inflammatory responses following infection. The endometrium is highly susceptible to SARS-CoV-2 cell entry; however, it remains unclear whether this could alter receptivity and embryo implantation. Menstrual cycle changes were reported in women who experienced severe infection; however, they tended to be reversible. For couples undergoing assisted reproduction treatment, the pandemic led to a significant psychological burden, with changes in lifestyle that could directly affect the success of the treatment. Human reproduction societies recommend screening all patients prior to cycle initiation and avoiding treatment of women with severe comorbidities until the pandemic is under control. Finally, for pregnant women, it is expected that the infection is more severe in women in the third trimester and in those with comorbidities. Those who are symptomatic for SARS-CoV-2 are more likely to have increased rates of prematurity and intrapartum fetal distress than those who are asymptomatic. Vertical transmission cannot be completely ruled out, but neonatal infection rates are low. Vaccination appears to be safe and is indicated for use in pregnant and lactating women because the benefits outweigh the risks.

## INTRODUCTION

The first case of severe acute respiratory coronavirus 2 (SARS-CoV-2) infection, which causes coronavirus disease 2019 (COVID-19), was reported on December 31, 2019, in Wuhan, China. Since then, it has rapidly spread worldwide, leading the World Health Organization (WHO) to declare it a pandemic on March 11, 2020 (1,2). Overall, more than 100 million cases have been detected worldwide, and over 2.5 million people have lost their lives to the disease.

Coronaviruses are an endemic in humans and cause 15-30% of yearly respiratory tract infections (1). In the past two decades, there have been outbreaks of SARS-CoV-1 (2002-2003) and the Middle-east respiratory syndrome coronavirus (2012), leading to 8000 and 2500 cases, respectively, with variable mortality rates (9% and 35%, respectively) (1).

Most cases of COVID-19 are mild and do not need medical assistance; however, 15-20% of the infected people require hospitalization and 5% required mechanical ventilation (2). Although the mortality rate is approximately 1%, the rapid spread of the infection, with a reproductive number (R_0_) of 2.2 (2), raises concerns about the risk of health care system collapse.

The major symptoms of the SARS-CoV-2 infection include fever, cough, myalgia, shortness of breath, headache, diarrhea, and anosmia. Severe cases lead to critical pneumonia and death. Risk factors for severe illness include age and comorbidities, such as cardiovascular disease, cancer, and chronic respiratory disease (3). Transmission is mainly via respiratory droplets, making social distancing one of the most efficient measures for the control of the pandemic.

For SARS-CoV-2 cell entry, the angiotensin-converting-enzyme 2 receptor (ACE2) is needed. The spike proteins (S-proteins) present in virus cell membranes bind to the ACE2 receptor, while the transmembrane serine protease 2 (TMPRSS2) present in the host cell, cleaves the S protein in its two domains (S1 and S2), allowing the membrane fusion and viral RNA entry in the host cell (3,4). Therefore, for viral infection both ACE2 and TMPRSS2 are needed (5).

Data regarding the effect of COVID-19 on reproductive outcomes is limited. Considering the recent outbreak, there is a need for further observation until permanent conclusions can be made. The presence of ACE2 receptor and TMPRSS2 in both male and female reproductive tract cells (1,3) has raised concerns that the SARS-CoV-2 infection may compromise human fertility and pregnancy.

In this paper, we aimed to summarize the available data related to the effects of the SARS-CoV-2 infection on the human reproductive tract and aspects related to COVID-19, assisted reproductive therapy, and pregnancy outcomes.

### Effects of SARS-CoV-2 on gametes

Although the male reproductive tract has a higher expression of ACE2 receptors than the female reproductive tract, both contain cells susceptible to viral infection. Spermatogonial, Sertoli, and Leydig cells are enriched with ACE2 and express TMPRSS2 as well, especially spermatogonia and spermatids (2). However, some studies show that the co-expression of ACE2 and TMPRSS2 is limited (5).

Liu et al. performed scRNA-seq in human adult testes (seven men with obstructive azoospermia and two healthy donors) and reported that TMPRSS2 is expressed at high levels in spermatogonial cells, while ACE2 is expressed at low levels (6). Sertoli cells have higher expression of ACE2 and lower expression of TMPRSS2. Zhao et al. and Whang et al. also reported that the expression of ACE2 in Leydig and Sertoli cells was almost three-fold higher when compared to spermatogonia (7,8).

One study analyzed semen samples of 34 men recovering from COVID-19 and did not detect the presence of SARS-CoV-2 in any ejaculated sample after a median of 31 days (9). Another cohort study performed semen testing on 38 men positive for SARS-CoV-2 and found that six of them had positive reverse transcription polymerase chain reaction (PCR) for the virus (four men in acute stage of disease and two in recovery) (10). However, the information regarding viral shedding or concentration in semen samples remains elusive.

In previous studies investigating SARS-CoV-2 RNA in the male reproductive tract, no viral material was detected in the prostatic fluid and 98% of the seminal fluid analyzed was negative for SARS-CoV-2 RNA. Although the details regarding the semen collection protocol and PCR kit were scarce, viral shedding was not demonstrated. Therefore, the authors agreed that these results should be confirmed by other studies before introducing any change in clinical practice (1). Song et al. analyzed ACE2 and TMPRSS2 co-expression in prostate cells and found that both receptors were present in less than 1% of the prostate cells (11), which is in accordance with previously published studies (1,5,9). Since viral shedding has not been found in semen, prostate, or seminal fluid and since viral transmission through intercourse or insemination has not been shown (for the disease to be considered sexually transmitted), there is currently no available evidence to consider COVID-19 a sexually transmitted infection. However, sexual contact in the acute stage of the disease can lead to partner contamination due to respiratory droplets (1).

Published cohort and case-control studies evaluating the presence of SARS-CoV-2 in the female reproductive tract found similar results (2,12). Further, over 98% of the vaginal fluid samples and all the cervical smears tested negative for the virus RNA (1). Cui et al. reported that all fluids tested were negative; however, the infection rate of the sexual partners was 42.9% (12). This supports the hypothesis that, although not considered a sexually transmitted infection, the intimacy of the sexual contact can transmit SARS-CoV-2 through respiratory droplets.

The ovaries have a gonadotropin-dependent expression of ACE2 receptors which is present in both pre-menopausal and post-menopausal women (2,3). Interestingly, Barragan et al. reported the absence of SARS-CoV-2 RNA in 16 oocytes from donors. The authors detected the presence of ACE2 in 5/16 oocytes but no expression of TMPRSS2 (13).

### SARS-CoV-2 and male fertility

There is limited data on the effects of SARS-CoV-2 on male fertility. It seems unlikely that gametes are affected due to the limited co-expression of ACE2 and TMPRSS2 (5). Some studies, however, have analyzed mechanisms that could influence male fertility following COVID-19. Xu et al. reported that *in situ* hybridization, morphological analysis, and immunohistochemical analysis were undertaken on the testes of six men who died from COVID-19 and the results were compared with those from the autopsy of four non-infected men. The authors found that the testes of affected men showed increased peritubular fibrosis, vascular congestion, and extensive germ cell destruction, but contained few spermatozoa within the seminiferous tubules and considerable inflammatory infiltrate (2,5,14).

Fever during meiosis and spermiogenesis may reportedly alter sperm motility and concentration (15). Therefore, it could be hypothesized that COVID-19 could, at least temporarily, affect spermatogenesis. However, male infertility is not commonly seen in areas where febrile diseases are endemic, such as malaria; therefore, the extent to which these observations are applicable to COVID-19 remains unknown.

The SARS-CoV-2 infection can lead to a cytokine storm and activate pathogenic pathways that may increase sperm DNA fragmentation and oxidative stress, contributing to a decrease in fertilizing potential (16). As early embryos express high levels of ACE2, a deleterious impact on embryo development cannot be completely ruled out. Ma et al. and Wang et al. both showed that the endocrine function in men affected by COVID-19 may be damaged due to lower levels of testosterone to luteinizing hormone (LH) ratio when compared to controls, which can reflect a compromised testicular function (17,18). These observations show that, at least after 3 months following infection, spermatogenesis and testicular function may be impaired.

It is unlikely that sperms are susceptible to infection, although inflammatory responses could alter the testis-blood barrier. However, testicular infection and orchitis has been observed, especially in elderly patients and in those with a more severe form of the disease; approximately 5-10% of the men of reproductive age have had infection of the testes (1).

### Sex susceptibility to SARS-Cov-2 infection

Epidemiologic studies have shown a susceptibility of male sex to more severe COVID-19 infection and higher mortality rates. This was believed to be due to the higher frequency of comorbidities, overall poorer health status, and lifestyle factors (smoking and sedentarism). However, some theories have been proposed to explain this susceptibility (5). One suggestion is that at first, the ACE2 expression is negatively regulated by the levels of estradiol (19). Androgens also play a role in the infection as transcriptional promoters for TMPRSS2 and in the up-regulation of this receptor. Therefore, women should have lower cellular expression levels of TMPRSS2 due to lower levels of circulating androgens (5).

A difference in expression of ACE2 receptors in both sexes can also lead to a higher susceptibility for infection in males. The ACE2 gene is located in the short arm of the X chromosome, a region that is more likely to escape from condensation into a Barr body and a cytologically detectable heterochromatic structure that results from the inactivation of one of the female X chromosomes. Females have two copies of ACE2, making it less likely for rapid viral saturation of receptors to occur, thus leading to less severe symptoms of COVID-19. Furthermore, the renin-angiotensin system (RAS), which is also regulated by ACE2, protects against vascular compromise and severe organ damage (5).

ACE2 is essential to deactivate the detrimental effects of the RAS. The pathway that culminates in angiotensin II formation leads to vasoconstriction, sodium reabsorption, and fluid retention to increase blood pressure. The ACE2 axis is a counterregulatory branch of the renin-angiotensin-system that converts angiotensin II into angiotensin 1-7, a peptide with anti-inflammatory, antifibrotic, and vasodilatory properties. The enhanced expression of ACE2 seen in females could, therefore, be another protective mechanism against vascular damage in severe disease (20).

### SARS-CoV-2 and the endometrium

The endometrium expresses low levels of ACE2 and high levels of TMPRSS2. However, other proteins and receptors are present in this tissue and can be related to SARS-CoV-2 infectivity. TMPRSS4 increases viral infectivity in the gut cells and is also present in the endometrium. Cathepsins B, L, and FURIN are other proteins that can cleave the “spike proteins” and mediate membrane fusion, while Myxovirus resistance 1 (MX1) favors infection through protein S modification (enzyme modifying protein structure) by neutrophil elastase. The basigin (BSG) receptor can also mediate SARS-CoV-2 binding to the host cell in pathway other than the one through the ACE2 receptor (21).

Castillo et al. performed a systematic review of literature to analyze the impact of the SARS-CoV-2 infection on the endometrium. They found that TMPRSS4, CTSL, CTSB, FURIN, (the latter three proteases), MX1, and BSG were highly expressed throughout the menstrual cycle, while TMPRSS2 expression was moderate and ACE2 was low. Except for TMPRSS2, all genes had significant changes across the menstrual cycle, with enhanced expression at the secretory phase. Whether this is implicated in endometrium receptivity and has a deleterious effect on embryo implantation is yet to be clarified (21).

### SARS-CoV-2 infection and the menstrual cycle

Viral infections can affect the female reproductive system and cause menstrual disturbances, as already demonstrated with hepatitis B and C viruses and HIV. Anovulation has been reported in acute diseases, probably related to transient ovarian function suppression to assure function of essential organs (22). Therefore, menstrual cycle changes because of the SARS-CoV-2 infection may be plausible.

Li et al. analyzed sex hormones levels and menstruation in a cohort comprising women of reproductive age hospitalized for COVID-19 (22). They divided patients into those with mild or severe symptoms and compared their hormonal levels to those of healthy women (controls) without ovulatory disturbances undergoing hormonal dosages for fertility treatment. Patients who experienced menstrual changes during the SARS-CoV-2 infection were more likely to have decreased menstrual volume and longer cycles (prolonged to 8-14 days). This difference, however, was not statistically significant when compared to women who had not experienced cycle changes or between those with mild or severe symptoms. After 3 months of follow up, their menstrual cycles had returned to normal. Sex hormones (FSH, LH, E2, progesterone, and testosterone) and the anti-Mullerian hormone concentrations also did not differ between women with COVID-19 and the controls (22). To date, there is no evidence that COVID-19 can affect the ovarian reserve; however, further studies are required to clarify this.

### Psychological impact of the COVID-19 pandemic in women with infertility

The COVID-19 pandemic severely affected infertility treatments. International societies (such as, the American Society for Reproductive Medicine [ASRM] and European Society for Human Reproduction and Embriology [ESHRE]) advised in the beginning of the pandemic to stop assisted reproductive treatments, except in cases of oncological fertility preservation (23,24). Moreover, many countries adopted a lockdown, limiting patients’ access to fertility care temporarily. This led to psychological distress and considerable changes in the health and lifestyle of patients undergoing fertility care.

Cirillo et al. evaluated the effects of the COVID-19 lockdown on the emotional health and lifestyles of women undergoing assisted reproductive treatment in a fertility center in Italy. They found that most women had considerable changes to their body mass index, with weight gain being the most common. There was also an increased in the consumption of red meat, sweets, sugared beverages, alcohol, and tobacco. Although women also increased their consumption of vegetables, fruits, and legumes, there was a decrease in adherence to the Mediterranean diet, that is considered a gold-standard of healthy diet in Italy. The higher consumption of snacks and sweets was most related to feelings of anxiety and boredom. Furthermore, most patients stopped exercising during this period. Women also reported increased levels of anger, sadness, and sleeping disturbances (25).

Lawson et al. reported data on psychological distress due to postponed fertility care in the pandemic. Patients were randomized to receive supplemental education explaining the rationale behind recommendations to delay fertility treatments due to the COVID-19 pandemic. Most women agreed with the recommendations to postpone fertility care. Yet, more than half of patients reported moderate to severe distress associated with cycle cancellation (26).

The preconceptional period is the time for women to adjust their lifestyle and control comorbidities. Therefore, changes in diet and exercise patterns could be deleterious to patients undergoing fertility care, and could enhance cardiovascular risk and lead to a worse response to ovarian stimulation. Psychological distress can also act as a modifiable risk factor, since most women reported changes in the consumption of snacks and sweets due to anxiety (25). The psychological impact of the COVID-19 pandemic can play an important role in worsening the outcomes for couples undergoing assisted reproduction treatment.

### SARS-CoV-2 and human reproduction

Since WHO declared COVID-19 a pandemic, the main international fertility societies [ASRM, ESHRE, International Federation of Fertility Societies (IFFS)] have published and updated several guidelines, recommendations, and bulletins to guide physicians worldwide in the management of fertility treatments (27). The common points initially addressed were to suspend initiation of reproductive treatments, including ovulation induction, intrauterine inseminations, *in vitro* fertilization, oocyte and sperm cryopreservation, and fresh/frozen embryo transfers (27). Medical appointments were advised to be provided via telemedicine as well (19).

After the most critical period, the societies unanimously agreed that infertility is considered a disease and human reproduction is an essential human right. Therefore, infertility treatment could be considered as an essential service. According to ARSM, ESHRE, and IFFS, as long as the efforts towards the control of SARS-CoV-2 spread succeeded, fertility care could be resumed (19).

Special recommendations should be made regarding clinical and laboratorial management. Patients with comorbidities such as severe obesity; uncontrolled diabetes; hypertension; previous organ transplant; immunosuppressive therapy; or hepatic, renal, or pulmonary diseases should be counseled to avoid initiation of treatment. Patient screening can be made with a checklist for symptoms or serology or PCR examinations. Mitigation measures should be considered depending on the incidence of infection, such as decreasing the number of patients treated, limiting access to treated patients only, limiting staff exposure, allowing more time between patient appointments, enhancing sanitation measures, increasing the use of telemedicine, avoiding embryo transfer, and advising a freeze-all strategy for all patients. All cycles should be cancelled if the patient tested positive for SARS-CoV-2, except in urgent cases, such as fertility preservation for oncological reasons. Laboratories require level 2 biological containment measures when dealing with SARS-CoV-2 positive material. Although evidence on the probability of cross contamination is lacking, it is advised to cryopreserve positive material in separate containers (19).

ASRM recommends that both pregnant women and women in the periconceptional period should be vaccinated. Assisted reproductive treatments should be avoided at least 3 days prior and 3 days after the vaccination (28). ESHRE, on the other hand, states that the decision to receive or decline the vaccine rests on the individual’s risk. Professional advice is strongly recommended both in pregnant women and those planning to conceive (29).

It is important to mention that no pregnant women were included in any vaccine trial and there is uncertainty about their safety during pregnancy and lactation. Although pregnancy was an exclusion criterion for vaccine studies, some women enrolled in the Pfizer-BioNTech study and the Janssen study did get pregnant after enrollment. The incidence of abortion was similar between the vaccine and placebo groups, although the sample size was small. The Food and Drug Administration concluded that both vaccines “did not have any adverse effects on female reproduction, fetal/embryonal development, or postnatal development” (30).

The AstraZeneca vaccine uses a viral vector (chimpanzee adenovirus vector) that has been modified to contain the gene encoding the spike protein of the SARS-CoV-2 (31). This technology has previously been used with the Ebola vaccine and has been proven safe when administered during pregnancy (32). Cases of thrombosis associated with thrombocytopenia have been reported after vaccination with the AstraZeneca vaccine and have been referred to as vaccine-induced immune thrombotic thrombocytopenia. However, these thrombotic events are rare in vaccinated individuals and have been fewer than that observed in the general population (31). It is also important to remember that the SARS-CoV-2 infection can lead to vascular damage, severe endothelial injury, and thromboembolic events associated with higher mortality rates in infected patients; therefore, the benefits of vaccination may outweigh the risks (31). Some countries, however, contraindicate the AstraZeneca vaccine during pregnancy. Considering the high estradiol levels during ovarian stimulation and its prothrombotic effect, more studies will be necessary to evaluate the safety of vaccination in women undergoing assisted reproductive treatment.

Vaccines also appear to be safe during lactation. The half-life of mRNA is short; thus, it is unlikely to enter breastmilk. Even if it does, it probably will be broken down during the digestive process (30). It is also important to remember that in the context of the potentially increased severity of COVID-19, emergence of new virus variants, and community transmission in countries where the pandemic is not under control, it is important to guarantee vaccine access to people who are at risk of worse outcomes if infected.

### SARS-CoV-2 and pregnancy outcomes

The physiological changes that occur during pregnancy, especially those involving the respiratory and cardiovascular systems, make pregnant women more prone to severe cases of respiratory tract infections, as observed in the H1N1 influenza pandemic (33). The beginning of the SARS-CoV-2 pandemic raised important concerns about COVID-19 and pregnancy outcomes for both mothers and fetuses. Large cohorts involving more than 23,000 pregnant women in the UK and USA reported that most patients have mild symptoms of the disease and the mortality rate in pregnancy is similar to that in the general population, at around 1%. Moderate and severe cases of COVID-19 seemed to be more common in the third trimester of pregnancy. In these studies, the transmission rate to newborns was low (33-35).

Hazari et al. performed a case-control study in Dubai including 77 SARS-CoV-2 infected pregnant women and 85 non-pregnant infected women and concluded that the frequency of symptoms was lower in pregnancy, but that the incidence of severe cases was higher and worse in women in the third trimester and in patients with comorbidities. Despite this, there were no statistical differences regarding mechanical ventilation, intensive care unit admission, systemic complications, and death. Moreover, there were no differences in neonatal outcomes when compared to the general population (33).

A multicentric cohort in Barcelona evaluated pregnant women in the second or third trimester affected by COVID-19 and compared them to a control group of non-affected pregnant women. The authors found that the COVID-19 incidence was similar during early and late pregnancy. There was a non-statistically significant trend of higher prematurity in pregnant women infected by SARS-CoV-2 and a significant higher incidence of intrapartum fetal distress in this group. Symptomatic cases were most common in the third trimester and the rates of prematurity, severity, and small for gestational age were higher in this group of patients when compared to the non-affected and asymptomatic affected women. There were no maternal deaths and no cases of vertical transmission in this study (36).

A study compared maternal and perinatal outcomes of pregnant women with COVID-19 at the time of birth between May 2020 and January 2021. The study included more than 340,000 women with singleton pregnancy, of whom 3,527 were recorded as having laboratory-confirmed SARS-CoV-2 infection. Fetal death, preterm birth, preeclampsia/eclampsia, and emergency cesarean delivery were significantly more common in infected women. Risk of neonatal adverse outcomes, need for specialist neonatal care, and prolonged neonatal admission following birth, were also higher for infants with mothers with laboratory-confirmed SARS-CoV-2 infection. For both mothers and infants, hospital readmission was significantly increased in the COVID-19 group (37). The link between COVID-19 and an increased risk of fetal death strengthens the recommendation that the benefits of vaccination in pregnant women outweigh the possible harm.

The pandemic led to adverse effects in perinatal and maternal outcomes related to reduced access to both health care and health care-seeking. A systematic review and meta-analysis that included 40 studies from 16 high-income and low or medium-income countries showed a higher incidence of maternal death, stillbirth, and ruptured ectopic pregnancy during the COVID-19 pandemic compared to that in the pre-pandemic period (38). The data highlights the important differences observed between richer and poorer countries. The higher incidence in maternal death and stillbirth is related only to low-income and medium-income countries, as is a 5-fold increase in hypertensive disorders of pregnancy. On the other hand, the study showed a reduction in preterm birth before 37 weeks of gestation in the high-income countries. These differences contrast the inequality observed in health care assistance worldwide, especially during the most critical months of the pandemic. The incidence of postnatal depression, maternal stress, and anxiety levels are unrelated to income classification and showed a statistically significant elevation, compared to the pre-pandemic period in all studies that analyzed this outcome (38). This draws attention to the psychological impact of the SARS-CoV-2 pandemic and the possible sequelae it will impose on mental health.

Similarly, a Canadian study on postpartum mental illness during the COVID-19 pandemic showed an increased rate of mental health visits for postpartum individuals in 2020 than was expected for the period, especially from April to November (39). This was seen in both primary and psychiatrist care for anxiety, depression, and substance use disorders. Increases in the visit rates were higher for individuals’ 0-90 days postpartum, which is the period of higher risk for postpartum mental illness. Postpartum individuals living in lower income neighborhoods showed smaller increases in visit rates, as was observed by Chmielewska et al. in their study (38,39), reflecting the unequal access to health care according to income.

A systematic review of 29 studies and 564 pregnant women with COVID-19 investigated the possibility of vertical transmission of SARS-CoV-2 to newborns. Of the 555 neonates included, 549 underwent the test for SARS-CoV-2, in which 18 (3.28%) tested positive. Four cases were tested for viral RNA on the mother's vaginal secretions, placenta, umbilical cord blood, amniotic fluid, and breast milk, with only one positive result in the amniotic fluid. Although the likelihood of postnatal infection is low, it could not be ruled out. In two cases, the diagnosis was made on the fifth day of life; thus, there could be the possibility of human-to-human transmission. Another two cases failed to be isolated immediately from their mothers and were observed unprotected in the operating room for 30 minutes; thus, the contaminated air in the operating room could be a source of pollution. Finally, only one case presented with high levels of IgG and IgM antibodies for SARS-CoV-2 2 hours after birth, although repeated PCR tests on nasopharyngeal swabs were negative. This led to the hypothesis that viruses that invade from the placenta may not multiply in the nasopharynx (40).

## CONCLUSION

Data on the SARS-CoV-2 infection is constantly changing and being updated since the beginning of pandemic. Until now, there is no consistent evidence in support of COVID-19 affecting the ovarian tissue or it being a sexually transmitted disease. Male fertility can be at least temporarily impaired by the inflammatory response that follows infection. Some women may have menstrual disturbances that tend to be reversible. The endometrial tissue expresses receptors and proteins involved in SARS-CoV-2 infectivity, but it remains unknown whether this can alter endometrial receptivity and embryo implantation. Regarding pregnancy, more severe cases tend to occur in women in the third trimester and in those with comorbidities, as in the general population. Reduced access to health care and reduced health care-seeking due to fear of infection also contributed to the higher incidence of adverse maternal and fetal outcomes. Neonatal infection rates are low; however, vertical transmission cannot be completely ruled out. Although vaccination appears to be safe and is indicated for use in pregnant and lactating women because the benefits outweigh the risks, more data is needed to provide definitive conclusions.

## AUTHOR CONTRIBUTIONS

Delamuta LC was responsible for the data analysis and manuscript writing. Monteleone PAA and Maciel GAR were responsible for the study design and manuscript review. Ferreira-Filho ES, Heinrich-Oliveira V, Soares-Júnior JM and Baracat EC were responsible for the manuscript review.
